# Improved Accuracy of Thermal Desorption Spectroscopy by Specimen Cooling during Measurement of Hydrogen Concentration in a High-Strength Steel

**DOI:** 10.3390/ma13051252

**Published:** 2020-03-10

**Authors:** Eric Fangnon, Evgenii Malitckii, Yuriy Yagodzinskyy, Pedro Vilaça

**Affiliations:** Advanced Manufacturing and Materials, Department of Mechanical Engineering, School of Engineering, Aalto University, 02150 Espoo, Finland; evgeny.malitskiy@aalto.fi (E.M.); yuriy.yagodzinskyy@aalto.fi (Y.Y.); pedro.vilaca@aalto.fi (P.V.)

**Keywords:** hydrogen, thermal desorption spectroscopy, sample cooling, high-strength steel, hydrogen concentration, hydrogen loss

## Abstract

Thermal desorption spectroscopy (TDS) is a powerful method for the measurement of hydrogen concentration in metallic materials. However, hydrogen loss from metallic samples during the preparation of the measurement poses a challenge to the accuracy of the results, especially in materials with high diffusivity of hydrogen, like ferritic and ferritic-martensitic steels. In the present paper, the effect of specimen cooling during the experimental procedure, as a tentative to reduce the loss of hydrogen during air-lock vacuum pumping for one high-strength steel of 1400 MPa, is evaluated. The results show, at room temperature, the presence of a continuous outward hydrogen flux accompanied with the redistribution of hydrogen within the measured steel during its exposure to the air-lock vacuum chamber under continuous pumping. Cooling of the steel samples to 213 K during pumping in the air-lock vacuum chamber before TDS measurement results in an increase in the measured total hydrogen concentration at about 14%. A significant reduction in hydrogen loss and redistribution within the steel sample improves the accuracy of hydrogen concentration measurement and trapping analysis in ferritic and martensitic steels.

## 1. Introduction

Hydrogen embrittlement is a significant concern for metallic materials in a variety of industrial applications [1,2,3]. Development of modern advanced high-strength steels (AHSS), minimizing their sensitivity to hydrogen embrittlement (HE) in the presence of small hydrogen concentrations, requires accurate measurements of hydrogen concentration and trapping in the materials. Currently, the most commonly used experimental techniques for hydrogen trapping analysis and concentration measurement in metallic materials include gas chromatography (GC) with thermal conductivity detector (TCD) and thermal desorption spectroscopy (TDS) [4,5,6,7,8]. GC and TDS hydrogen measurement are both based on the method of hot extraction of hydrogen; however, their operation temperatures and measurements approaches are different [9,10,11]. TDS enables characterization of the hydrogen diffusion, trapping, and total hydrogen concentration measurements. Often, specific features of the TDS spectra are associated with the hydrogen trapping at dislocations, vacancies, vacancy complexes, grain boundaries, and interfaces of non-metallic inclusions (NMI), enabling the measurement of the hydrogen trapping activation energies [10,12,13].

A number of technical solutions comprising hot extraction in the ultra-high vacuum chamber of the TDS apparatus have been employed for the characterization of hydrogen in steels. Typically, the measurement apparatus is equipped with an air-lock vacuum chamber (AVC) for specimen transportation to ultra-high vacuum (UHV) chamber followed by hydrogen measurement [7,14,15]. For example, to study hydrogen in metals using TDS in [14], the authors placed the studied sample in a quartz tube equipped with a mass spectrometer. The quartz tube was pumped directly to the desired UHV pressure, followed by the measurement. In contrast to the setup adopted in [14], other authors in [2,7] used a combination of primary AVC for specimen insertion and pre-pumping, and a UHV chamber that operates in standby mode always under continuous pumping. In order to reduce the UHV pumping time and keep the measurement chamber free of contaminants, the specimen is first kept in the AVC. The AVC is pumped to an intermediate pressure of about 10^−4^ Pa, followed by the specimen transport into the UHV chamber equipped with a furnace and a quadrupole mass spectrometer (QMS) for measurement of desorbing hydrogen (see Figure 2) [2,7]. Steels are often exposed to hydrogen-containing environments, during production and in-service [16,17,18]. The control of hydrogen concentrations and trapping within the sample before measurement is a complicated task requiring proper sample extraction, delivery, and preparation procedures prior to hydrogen concentration measurements. The loss of hydrogen during sample transportation from the service environment to the measurement site can be reduced by storing the specimen in liquid nitrogen [19]. However, the problem of hydrogen loss in the course of TDS measurement preparation, namely during AVC pumping, has not been considered.

**Figure 1:**
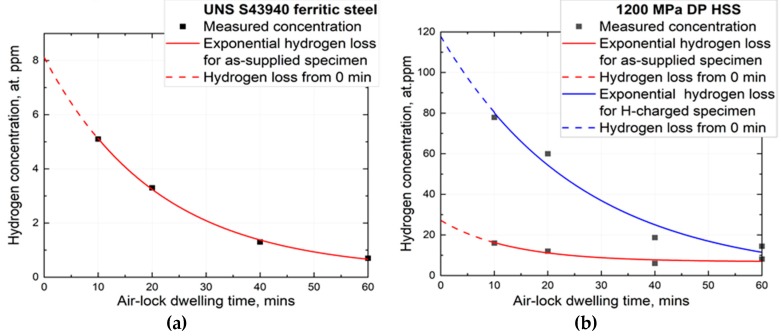
Hydrogen loss as a function of air-lock vacuum chamber (AVC) dwelling time for 18% chromium ASTM UNS S43940 ferritic stainless steel (**a**) and for as-supplied and hydrogen charged ferritic-martensitic HSS (**b**) [20].

Loss of hydrogen in AVC has been studied by Fangnon et al. [20]. For 18% chromium ASTM UNS S43940 ferritic stainless steel, the hydrogen loss during 15 and 60 min of AVC pumping is approximately 6.6% and 16% of the measured residual hydrogen concentration, respectively, as shown in Figure 1a.

Hydrogen diffusivity depends on temperature, hydrogen concentration, and material microstructure [21]. For example, the diffusivity of hydrogen in ferritic-martensitic HSS is relatively high as compared to ferritic and austenitic stainless steels [21,22]. Hydrogen loss in hydrogen-charged specimens is even more pronounced in comparison with non-charged specimens [20]. Figure 1b shows the hydrogen loss from as-supplied and hydrogen-charged specimens of a ferritic-martensitic HSS. Hydrogen losses after 60 min dwelling in AVC for as-supplied and hydrogen-charged HSS were calculated to be about 61% and 136% of their residual hydrogen concentration, respectively. The results show, however, that about 35–50% of total concentration was lost during the first 15 min of AVC dwelling [20].

Storage of specimens at cryogenic temperatures is used to suppress hydrogen diffusivity, preventing hydrogen loss in steel specimens prior to measurements. The process described in standard BS ISO 3690 is used to store samples up to 15 days before the hydrogen measurement [19,23,24]. In order to study the hydrogen diffusivity and trapping at low temperatures, the cryogenic-TDS technique (C-TDS) was developed [25,26]. Use of C-TDS for measurement of hydrogen in Fe-C alloy revealed the hydrogen thermal desorption starting at 173 K to room temperature (RT), associated preferably with the hydrogen trapping in solid solution, at dislocations and grain boundaries [27]. During AVC dwelling time at RT, hydrogen trapped at these trapping sites can diffuse to the surface of the specimen and leave, affecting the accuracy of the hydrogen concentration measurement performed by traditional techniques. The aim of this paper is to study the hydrogen loss during AVC pumping time and evaluate the effectiveness in minimizing this phenomenon, with the use of a specimen cooling system integrated in the AVC before TDS measurement.

## 2. Materials and Methods

A hot-rolled sheet of martensitic HSS with a tensile strength of 1400 MPa supplied by Voestalpine AG was selected for the study [28]. The chemical composition of the studied steel as provided by the manufacturer is shown in Table 1. The specimens were cut using electro-discharge machining and polished mechanically, finishing with #1200-emery paper. The size of the TDS specimens was 1 mm × 5 mm × 15 mm. The specimens were hydrogen-charged electrochemically in 1N H_2_SO_4_ solution with 10 mg/L of thiourea (CH_4_N_2_S) at a constant applied potential of −1.225 V for one hour. After charging, hydrogen content was measured using the TDS technique.

**Figure 2:**
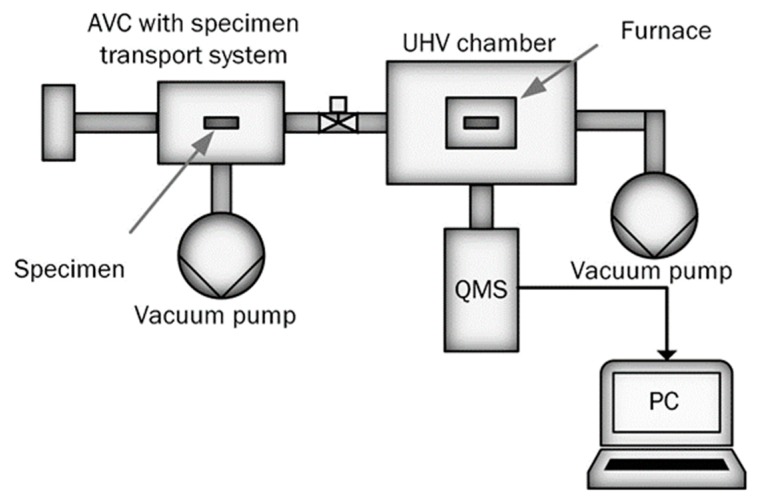
Schematic view of the thermal desorption spectroscopy (TDS) apparatus.

**Figure 3:**
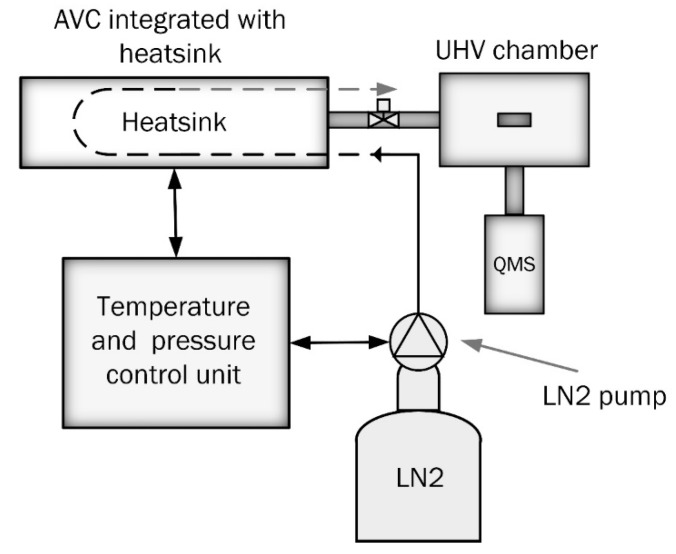
Schematic view of the AVC-coupled specimen’s cooling system.

Figure 2 shows a schematic view of the TDS apparatus (manufacturer, city and country) used in the study. The TDS apparatus was conceptualized, designed, and built at Aalto University in Espoo, Finland. The mass spectrometer and vacuum pumps were purchased from Agilent technologies, California, United states. The UHV measurement chamber is always kept under continuous vacuum pumping at 5 × 10^−7^ Pa, while the AVC is used for the specimen exchange and preliminary vacuum pumping before the transfer of the specimen to the measurement chamber. The preliminary pumping takes about 15 min to approach an intermediate pressure of about 10^−4^ Pa needed for safe and contamination-free transportation of the specimen to the UHV measurement chamber [2,7]. The time of preliminary pumping in AVC, we will call “AVC dwell time”. In order to reduce the hydrogen loss during AVC dwelling time, the AVC is equipped with a specimen’s cooling system. A heatsink was designed and fitted to the specimen holder providing heat transfer from the specimen. The schematic view of the AVC-coupled specimen’s cooling system is shown in Figure 3. Liquid nitrogen is used as the cooling agent. The heatsink temperature is controlled by an automatic electronic unit, which provides a specimen cooling rate of about 10 K/min with an accuracy of ±2 K.

**Figure 4:**
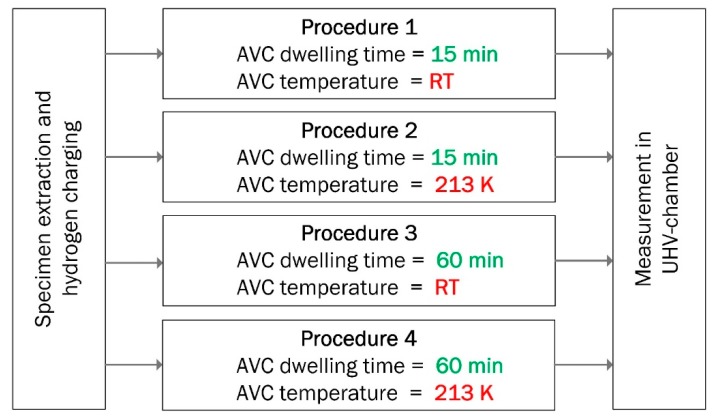
Experimental plan of the TDS measurements.

The AVC instrumented with the specimen’s cooling system was used to cool the specimens down to 213 K. Cooling continued for the entire AVC dwelling time until the specimen is transferred into the UHV chamber for measurement. All the TDS measurements were performed with a constant heating rate of 10 K/min in the temperature range from room temperature (RT) to 1070 K. Hydrogen concentration in the steel specimens was calculated by integrating the area under the TDS curve [28,29]. Hydrogen thermal desorption measurements were performed according to the procedure presented in Figure 4.

## 3. Results and Discussion

TDS measurements were performed to evaluate the effect of the specimen’s temperature and AVC’s dwelling time on the hydrogen concentration in the studied steel.

**Figure 5:**
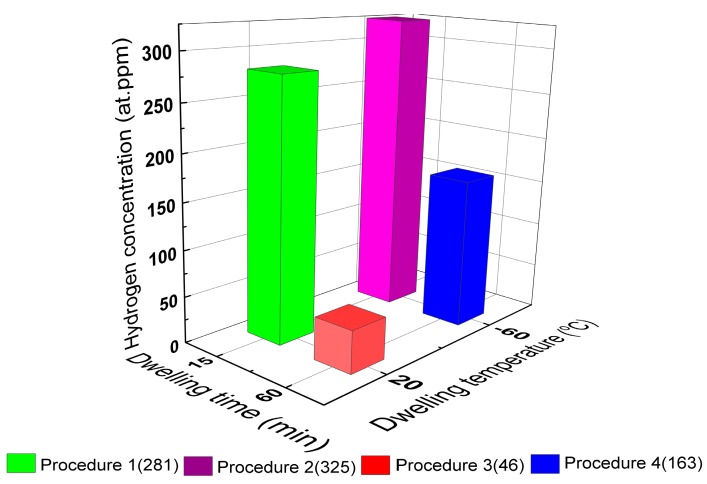
Hydrogen concentration (at.ppm) as a function of the AVC’s dwelling time and specimen’s cooling temperature.

As depicted in Figure 5, the total concentration of hydrogen measured for the AVC’s temperature at RT ≡ 293 K (procedure 1) and 213 K (procedure 2) was of about 281 at.ppm and 325 at.ppm, respectively. A relative reduction of about 14% was measured in procedure 1. With the increase of the AVC’s dwelling time, the concentration of hydrogen at RT ≡ 293 K (procedure 3) and 213 K (procedure 4) was calculated to be about 46 at.ppm and 163 at.ppm, respectively. A relative reduction of 72% was measured in procedure 3. Considering the coupled effects of AVC’s temperature and AVC’s dwelling time, the relative reduction of hydrogen concentration from the best condition, obtained for the procedure 2 (AVC’s temperature = 213 K and AVC’s dwelling time = 15 min), i.e., considering the 325 at.ppm = 100% was: 14% for the procedure 1 (281 at.ppm); 50% for the procedure 4 (163 at.ppm); and 86% for the procedure 3 (46 at.ppm). The results confirm the known detrimental effect of the AVC’s dwelling time in the hydrogen concentration [15,20]. Therefore, minimizing the AVC’s dwelling time is very important. The most important result is that the AVC’s temperature does not only improve the accuracy of the measurements for the same AVC’s dwelling time but also plays a very relevant role in reducing the sensibility of the TDS measurements to the AVC’s dwelling time. In fact, comparing the procedures 2 and 4, with same AVC’s temperature = 213 K, the increase of AVC’s dwelling time from 15 min to 60 min lead to loss of hydrogen of about 50%, versus a loss of about 84% in the total concentration of hydrogen, for the AVC’s temperature = RT ≡ 273 K, when comparing procedures 1 and 3.

**Figure 6:**
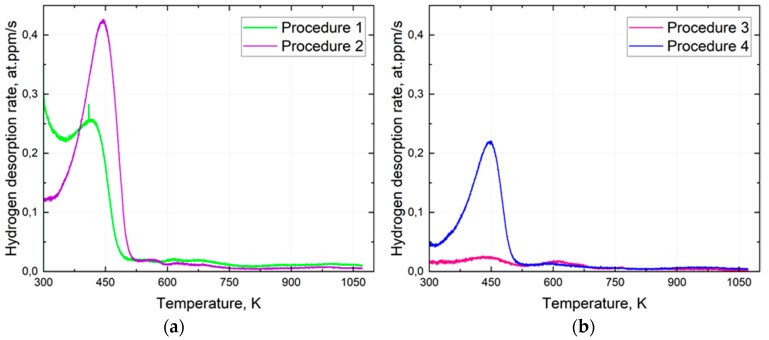
TDS curves corresponding to measurement procedures 1, 2, with the same AVC dwelling time = 15 min (**a**) and procedures 3, 4, with the same AVC dwelling time = 60 min (**b**).

**Figure 7:**
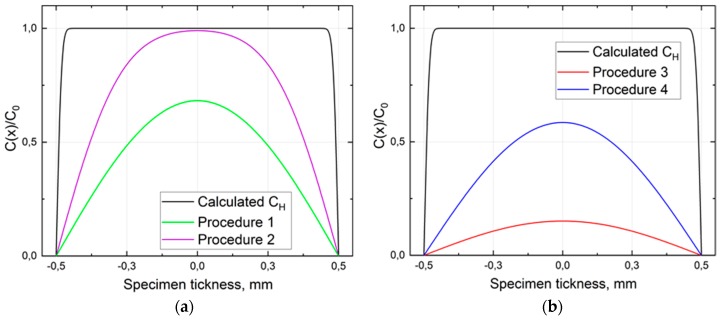
Concentration profiles of the calculated hydrogen concentration after one hour of electrochemical charging and 15 min AVC’s dwelling at room temperature (RT) and 213 K (**a**) 60 min AVC’s dwelling at RT and 213 K (**b**).

The hydrogen redistribution in the steel was studied by the hydrogen TDS analysis. The results of the TDS performed according to the measurement procedures 1 and 2 evidence a significant hydrogen redistribution within the sample material during the AVC’s dwelling time of about 15 min. As shown in Figure 6a, the initial hydrogen desorption rate measured at RT is almost three times smaller in pre-cooled specimens compared to those measured without cooling. The dynamic of the hydrogen thermal desorption rate change is also different for measurement procedures 3, and 4 caused apparently by a different distribution of hydrogen between the trapping sites of the studied steel. Cooling of the specimen holder to 213 K is obviously insufficient to suppress the diffusion of hydrogen fully in the studied steel (see Figure 7). Nevertheless, the use of the AVC-coupled specimen cooling system allows us to reveal the hydrogen trapping change caused by the hydrogen redistribution at RT and estimating the initial hydrogen distribution among the trapping sites. Slowing down the redistribution of hydrogen within the studied sample by cooling it can be observed by estimating the hydrogen concentration profile at each measurement. The change in the hydrogen concentration of interstitial solute atoms at a given point in a metallic material matrix x and the rate of diffusion are controlled by a diffusion process that is expressed by Equation (1) [29].

(1) $$D=\frac{x^{2}}{\alpha^{2}t}$$

where α is a constant, depending on the concentration difference of the solute atoms at a given point within the material matrix, and t is the charging time. Electrochemically charging the studied steel sample to saturation in one hour at RT, the effective diffusivity is calculated with equation (1) to be D = 2.78 × 10^−10^ m^2^s^−1^. An expression (2) for hydrogen concentration profiles at distinctive dwelling times and temperatures can be obtained for a thin plate by solving the equation of the second Fick’s law [7].

(2) $$C\left(x\right)=\frac{4C_{0}}{\pi }\overset{\infty }{\underset{n=0}{\sum }}\frac{{\left(-1\right)}^{n}}{\left(2n+1\right)}cos\frac{\left(2n+1\right)\pi x}{h}\left(1-e^{-\frac{\pi^{2}{\left(2n+1\right)}^{2}D\left(T_{s}\right)t_{s}}{h^{2}}}\right)e^{-\frac{\pi^{2}{\left(2n+1\right)}^{2}D\left(T_{d}\right)t_{d}}{h^{2}}}$$

where $C_{0}$ is the concentration of hydrogen on the specimen surface, $t_{s}$ and $T_{s}$ are time and temperature of hydrogen charging, $t_{d}$ and $T_{d}$ are time and temperature of hydrogen desorption, respectively, *h* is the thickness of the specimen, and *D* is the diffusion coefficient, calculated from (1). The total hydrogen concentration immediately after electrochemical charging is estimated to be about 379 at.ppm. That is about 35% and 17% more than the concentration measured in procedures 1 and 2, respectively. Figure 7 shows the hydrogen concentration profiles for the studied steel immediately after charging, AVC’s dwelling for 15 min, and 60 min in AVC’s temperature at RT and 213 K. The area between the profile curves corresponds to hydrogen loss from the sample.

**Figure 8:**
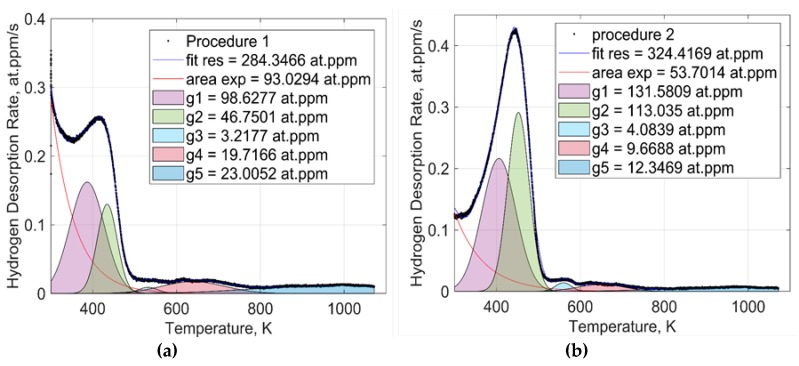
Fittings of the TDS curves measured according to procedures 1 (**a**) and 2 (**b**).

The TDS curves of the hydrogen release from the studied steel obtained according to measurement procedures 1 and 2 have a complex shape caused by a considerable amount of the different trapping sites for hydrogen. The hydrogen desorption rate is apparently dependent on the initial hydrogen distribution between the trapping sites. The obtained spectra have a well-defined low and high temperature components from RT to 600 K and from 600 K to 800 K, respectively. The complex character of trapping was analyzed assuming that the low-temperature component of the curve consists of a few Gaussian peaks, as was suggested by Smith et al. [30]. The TDS curves fitted with the Gaussian peaks are shown in Figure 8. It is assumed that the exponential background can be associated with a combination of two phenomena. One is the so-called “diffusible hydrogen” (DH), which is characterized by a high diffusion rate at RT [27]. The diffusion rate reduces exponentially with the reduction of hydrogen in thesolid solution of the steel. The other is the flooding of hydrogen from the AVC to the UV chamber during specimen transport. The low-temperature component of the TDS curves can be fitted as the sum of three Gaussian peaks and one exponential background curve. Hydrogen concentration was calculated from the fitting separately for each Gaussian peak and exponential background, as shown in Figure 8.

Figure 8a,b summarizes the hydrogen concentrations calculated separately for each Gaussian peak of the TDS curves obtained by measurement procedures 1 and 2, respectively. The hydrogen concentration associated with the exponential background of the TDS curve obtained in procedure 1 is almost 2 times smaller compared to that obtained in procedure 2. Peaks 1 and 2 of the pre-cooled specimens (procedure 2) has more hydrogen concentration compared to their counterpart measured after AVC’s dwelling time at RT (procedure 1). The pre-cooled specimen, peaks 1, 2, and 3 are shifted to higher temperatures (420 K, 470 K and 560 K, respectively) compared to that in specimen measured after AVC’s dwelling time at RT (370 K, 430 K, and 540 K, respectively). Peak 3 obtained for both measurement procedures do not show a significant difference in hydrogen concentration (3 at.ppm and 4 at.ppm after measurement procedures 1 and 2, respectively).

**Figure 9:**
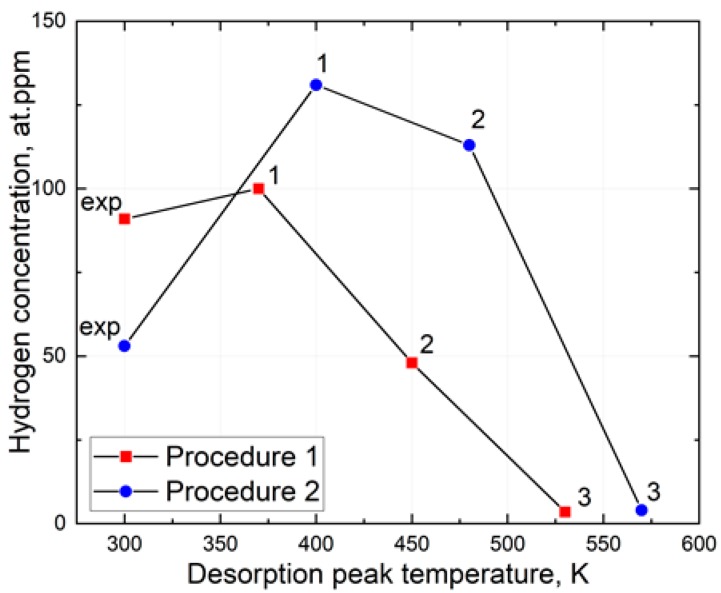
Summarized data of the hydrogen concentration calculated separately for each Gaussian peak and exponential background of the TDS curve obtained by measurement procedures 1 and 2.

The observed complex hydrogen thermal desorption spectra are related to the microstructure of the studied steel and hydrogen distribution between trapping sites. The increase of the total hydrogen concentration observed in the measurement procedure 1 is accompanied by the growth of the low-temperature component rather than the high-temperature component of the thermal desorption spectra. The increase in the lower temperature component (300 K) of procedure 1 can be associated with the flooding of hydrogen from the AVC to the UHV chamber during specimen transport. One can assume that the high-temperature component of the TDS spectra is associated with the decomposition of the molecular hydrogen trapped in voids and bubbles during the metallurgical processes. Another possible reason is the decomposition of the hydrocarbon deposits on the surface of the specimen [31]. The assumptions need, however, further investigation. Fitting procedure applied to the TDS spectra is targeted to characterize the trapping sites of hydrogen in the studied steel. Hydrogen trapping energy associated with a specific trapping sites like vacancy and vacancy complexes, NMI interfaces, retained austenite inclusions, etc., has a broad distribution that usually allows to link a certain Gaussian peak to a specific trapping site [30]. One can observe from Figure 9, the peaks 1, 2, and 3 are shifted to low temperature for procedure 1 compared to that for procedure 2 at about 40 K. Such a phenomenon controlled apparently by hydrogen diffusion in solid solution and total hydrogen concentration. At high hydrogen concentrations, hydrogen thermal desorption rate approaches its maximum at about 0.43 at.ppm/s and shifts to the higher temperatures, as shown in Figure 6a. The assumption supported by the fact that at lower hydrogen concentration, the TDS peak temperature shift almost disappears as shown in Figure 6b. The concentration of hydrogen associated with peak 3 was calculated to be almost the same for measurement procedures 1 and 2. One may say that the cooling of the specimen in AVC does not affect the hydrogen redistribution significantly from peak 3 to peak 5 that relate apparently to hydrogen desorption from the retained austenite bulk [28,32,33]. Cooling of the specimen during the AVC dwelling time results in a significant increase in the hydrogen concentration in the trapping sites related to peaks 1 and 2, while the concentration of DH decreases markedly (see Figure 9). Higher concentrations relating to peaks 1 and 2 after measurement procedure 2 are associated with a reduction in the hydrogen desorption rate from the corresponding trapping sites of the crystal lattice defects or NMI and retained austenite interfaces [34,35]. Nevertheless, AVC specimen cooling does not affect significantly the diffusion of hydrogen in solid solution. Reduction of exponential background in procedure 2 can be associated with reduction of the hydrogen redistribution from the trapping sites of peak 1 and 2 into the solid solution.

Worth to note is that the maximum hydrogen thermal desorption rates observed at the peak of the low-temperature component of the TDS spectra measured without specimen-cooling (procedures 1 and 3) are comparable with that observed at RT. One can assume that the TDS spectra shape difference between procedure 1 and 2 is a result of the reduction of continuous outward diffusion and desorption of DH followed by trapped hydrogen associated with peak 1 and 2, creating a balance of hydrogen flux between hydrogen trapping. DH is associated apparently with hydrogen trapped at grain boundaries and dislocations [25]. Hydrogen relating to the peaks 1 and 2 is probably associated with hydrogen trapped at vacancies and vacancy complexes [36]. In other words, during AVC dwelling time, DH trapped in the solid steel solution diffuses to the surface of the specimen and leave following with a redistribution of hydrogen within the steel. Consequently, the hydrogen loss in the AVC affects the measurement of the total hydrogen concentration in steels depending on the studied steel microstructure, dwelling time, and temperature. Cooling of the specimen during the AVC dwelling time inhibits the detrapping and diffusion of DH, hence minimizes the redistribution of hydrogen within the steel.

## 4. Conclusions

The hydrogen loss in the studied high-strength steel during AVC’s dwelling time before hydrogen measurement with TDS was studied. An AVC-coupled cooling system was designed to improve the accuracy of the TDS measurement by reducing the hydrogen loss and redistribution of hydrogen within the steel before the measurement. The following conclusions were reached.

- The hydrogen loss showed to depend significantly on the AVC’s dwelling time and AVC’s temperature of the specimen.
- Cooling of the studied high-strength steel specimens at 213 K using the AVC-coupled cooling system resulted in an increase of the total measured hydrogen concentration at about 14% and 72% for 15 and 60 min AVC’s dwelling time, respectively, compared to those measured at RT.
- Cooling of the studied high-strength steel specimen results in a change in shape and position of hydrogen thermal desorption peaks.

Worth to note is that the TDS-coupled cooling system operating at 213 K cannot suppress fully the diffusion of hydrogen in ferritic-martensitic steels; however, it improves the accuracy of measured hydrogen concentration.

## Figures and Tables

**Table 1:** Chemical composition of the studied martensitic HSS (in wt%).

C	Si	Mn	Cr	Ti	N	Al	P	S	Fe
0.157	0.19	2.24	0.46	0.002	0.006	0.053	0.011	0.001	Balance
